# Association of viral hepatitis and bipolar disorder: a nationwide population-based study

**DOI:** 10.1186/s12967-018-1542-3

**Published:** 2018-06-22

**Authors:** Lee-Won Chong, Chih-Chao Hsu, Chang-Yin Lee, Ruey-Hwang Chou, Cheng-Li Lin, Kuang-Hsi Chang, Yi-Chao Hsu

**Affiliations:** 1 Division of Hepatology and Gastroenterology, Department of Internal Medicine, Shin Kong Wu Ho-Su Memorial Hospital, Taipei, Taiwan; 2 School of Medicine, Fu-Jen Catholic University, New Taipei City, Taiwan; 3 Division of Psychiatry, Taitung Branch, Taipei Veterans General Hospital, Taitung, Taiwan; 4 College of Medicine, The School of Chinese Medicine for Post Baccalaureate, I-Shou University (Yancho Campus), Kaohsiung, Taiwan; 5 Department of Chinese Medicine, E-DA Hospital, Kaohsiung, Taiwan; 6 Department of Chinese Medicine, E-DA Cancer Hospital, Kaohsiung, Taiwan; 7 Graduate Institute of Biomedical Sciences and Center for Molecular Medicine, China Medical University, Taichung, Taiwan; 8 Department of Biotechnology, Asia University, Taichung, Taiwan; 9 Management Office for Health Data, China Medical University Hospital, Taichung, Taiwan; 10 Department of Medical Research, Tungs’ Taichung Metroharbor Hospital, Taichung, Taiwan; 11 Graduate Institute of Biomedical Sciences, China Medical University, Taichung, Taiwan; 12 Institute of Biomedical Sciences, Mackay Medical College, New Taipei City, Taiwan

**Keywords:** HBV, HCV, Bipolar disorder, NHIRD

## Abstract

**Background:**

Bipolar disorder (BD), a type of psychiatric mood disorder, is manifested by chronic and recurrent mood fluctuations. This study aims to determine whether hepatitis B virus (HBV) or hepatitis C virus (HCV) infection is a risk factor for BD.

**Methods:**

A total of 48,215 patients with newly diagnosed viral hepatitis from 2000 to 2010 were identified and frequency-matched with 192,860 people without hepatitis. Both groups were followed until diagnosis with BD, withdrawal from the national health insurance program, or the end of 2011. Patients with viral hepatitis were grouped into 3 cohorts: HBV infection, HCV infection, and HBV/HCV coinfection. The association between viral hepatitis and BD were examined using Cox proportional hazards regression models.

**Results:**

The incidence of BD was higher in HBV/HCV coinfection than in the control group, with an adjusted hazard ratio of 2.16 (95% confidence interval 1.06–4.41) when adjusted for sex, age, and comorbidity. After further adjustment, we noted that an age more than 65 years and female may be associated with an increased risk of BD in patients with chronic hepatitis B and C.

**Conclusion:**

Viral hepatitis may be associated with increased risk of subsequent BD.

**Electronic supplementary material:**

The online version of this article (10.1186/s12967-018-1542-3) contains supplementary material, which is available to authorized users.

## Background

Viral hepatitis is the major cause of hepatitis [1]. Among all types of viral hepatitis, hepatitis B virus (HBV) and hepatitis C virus (HCV) entail infections that are known for engendering chronic liver inflammation and hepatic malignancy [2]. In addition to HBV or HCV monoinfection, HBV/HCV coinfection has been shown to be associated with an increased risk of disease progression and malignancy [3]. Moreover, it has been reported that hepatic inflammation plays a major role in HBV- or HCV-induced liver damage, and immune cells including CD8(+)T lymphocytes as well as proinflammatory cytokines are also involved in this damage [4]. Although hepatic inflammation is a symptom of HBV and HCV infection, the inflammatory response could be systemic, because of the immune reaction induced by the translocation of microbial products. [5]. Research has shown that HBV can replicate in neuronal cells, which may account for the neuropathy associated HBV infection [6]. Furthermore, HCV-infected microglia cells and astrocyte are detected in the brain, indicating biological basis of neurocognitive abnormalities in HCV infection [7]. Recently, four independent studies indicate that viral hepatitis is associated with subsequent Parkinson disease [8–11]. These papers raised a specific aspect of viral hepatitis, but whether this reflects shared disease mechanisms in genetic or environmental susceptibility, or sequelae of viral hepatitis per se, or a consequence of treatment remains to be determined. It is of great interest to note that the association between viral hepatitis and psychiatric diseases has not been explored yet. We therefore aimed to investigate the association between viral hepatitis and bipolar disorder.

Bipolar disorder (BD), a type of psychiatric mood disorder, is manifested by chronic and recurrent mood fluctuations and involves a spectrum of symptoms including depressive, hypomanic, and manic manifestations [12]. In the BD population, all-cause and suicide mortality rates are substantially approximately 2-fold and 10-fold higher, respectively, than those in the general population [13]. Osby et al. further reported that the increased death rate in patients with BD is partly attributable to various potentially life-threatening medical comorbidities, particularly cardiovascular diseases [14]. In a review of the evidence, Rege and Hopkinson suggest that systemic inflammation and neuroinflammation may be involved in the pathogenesis of BD [15]. Inflammatory cytokines such as tumor necrosis factor-α (TNF-α) and soluble tumor necrosis factor receptor type 1 have been reported to be involved in the inflammatory state [16]. Another hypothesis of the association between medical conditions and subsequent BD was through inflammatory process, such as rheumatoid arthritis [17], peptic ulcer disease [18], and gastro-esophageal reflux disease [19]. As there is a high prevalence of HBV or HCV infection in Taiwan, we are therefore interested in investigating whether chronic HBV or HCV infection is a risk factor for the development of BD. 

In treatment of hepatitis, interferon-α, one of the treatment of HBV and HCV, was reported having neuropsychiatric side-effects [20]. Also, in the treatment of chronic HCV infection, combination of interferon and ribavirin was reported having psychiatric adverse events, including major depression [21]. Additionally, a study conducted by Quarantini and colleagues demonstrated that patients with HCV infection exhibit cognitive impairment, especially over visuo-spatial memory performance, which may be specific to HCV infection rather than secondary psychiatric comorbidities [22]. Even though a case report demonstrated interferon therapy could induce mania [23], limited evidence investigated the association between HBV/HCV and subsequent BD. To test these hypothesis that HBV or HCV infection is associated with subsequent BD, we conducted a nationwide, population-based cohort study to investigate whether HBV or HCV infection increases the risk of BD.

## Methods

### Data source

The study was conducted using data from the Longitudinal Health Insurance Database (LHID), a data set comprising the claims data of people enrolled in the Taiwan National Health Insurance (NHI) program. The National Health Research Institutes (NHRI) randomly selected 1 million insured people from 1996 to 2000 and followed them. According to the NHRI report, the demographic characteristics showed no difference between the people in the LHID and those enrolled in the NHI program. The claims data in the LHID include a beneficiary registry, inpatient and outpatient files, and other medical services. All patient histories were collected from inpatient and outpatient files and recorded according to the International Classification of Diseases, Ninth Revision, Clinical Modification (ICD-9-CM). The NHRI released the database with anonymous patient identification numbers to protect the privacy of the insured people.

### Study population

This was a retrospective, population-based cohort study, and we included a cohort of hepatitis patients and a comparison cohort to observe the occurrence of BD. The hepatitis cohort comprised patients with new onset HBV (ICD-9-CM 070.20 Viral hepatitis B with hepatic coma, acute or unspecified, without mention of hepatitis delta; 070.22 Viral hepatitis B with hepatic coma, chronic, without mention of hepatitis delta; 070.30 Viral hepatitis B without mention of hepatic coma, acute or unspecified, without mention of hepatitis delta; 070.32 Viral hepatitis B without mention of hepatic coma, chronic, without mention of hepatitis delta, and V02.61 Hepatitis B carrier) or HCV (ICD-9-CM 070.41 Acute hepatitis C with hepatic coma; 070.44 Chronic hepatitis C with hepatic coma; 070.51 Acute hepatitis C without mention of hepatic coma; 070.54 Chronic hepatitis C without mention of hepatic coma, and V02.62 Hepatitis B carrier) from January 1, 2000 to December 31, 2010. The date of first diagnosis of hepatitis was defined as the index date. The comparison cohort contained patients in the LHID without a diagnosis of hepatitis. Each patient in the hepatitis cohort was randomly frequency-matched with 4 controls according to various criteria including age (per 5 years) and sex. For the index date of the comparison cohort, patients were assigned the same date as those of the matched cases. Both cohorts excluded patients without complete characteristic data and aged younger than 20 years or those with a history of BD before the index date. In addition, considering the undiagnosed mood symptoms, we also excluded the patients having depression (ICD-9-CM 296.2 Major depressive disorder, single episode; 296.3 Major depressive disorder, recurrent episode; 311 Depressive disorder, not elsewhere classified) before the index date. Moreover, HBV/HCV can be contracted during sex [24] and unsafe drug use [25]. Hence, before the index date, we excluded patients having the diagnosis of borderline personality disorder (ICD-9-CM 301.83 Borderline personality disorder), which has been associated with risky sexual behaviors (no using condoms with changed partners) and substance use problems [26], and we also excluded the patients having opioid dependence (ICD-9-CM 305.5 opioid abuse; 304.0 opioid type dependence). It has been suggested that the exclusion of these patients could decrease the selection bias. We followed these 2 cohorts until withdrawal from the insurance program, BD occurrence (ICD-9-CM 296 Episodic mood disorders), or December 31, 2011. The confounding factors in this study were age, sex, and BD-associated comorbidities. Sex difference was noted among hepatitis infection and may influence the outcome [27]. Patients with comorbidities were defined as those with a history of comorbidities before the index date. The BD-associated comorbidities in this study included cirrhosis (ICD-9-CM 571.2 571.2 Alcoholic cirrhosis of liver; 571.5 Cirrhosis of liver without mention of alcohol; 571.6 Biliary cirrhosis), hypertension (ICD-9-CM 401-405 Hypertensive disease), hyperlipidemia (ICD-9-CM 272 Disorders of lipoid metabolism), asthma (ICD-9-CM 493 Asthma), coronary artery disease (CAD, ICD-9-CM 410-414 Ischemic heart disease), alcohol-related disorder (ALD, ICD-9-CM 291 Alcohol-induced mental disorders; 303 Alcohol dependence syndrome; 305.0 Alcohol abuse; 571.0 Alcoholic fatty liver; 571.1 Acute alcoholic hepatitis; 571.3 Alcoholic liver damage, unspecified; 790.3 Excessive blood level of alcohol; A215, and V11.3 Alcoholism), anxiety [28] (ICD-9-CM 300.00 Anxiety state, unspecified). Additionally, we listed major depressive disorder (ICD-9-CM 296.20 Major depressive disorder, single episode; 296.30 Major depressive disorder, recurrent episode; 311 Depressive disorder, not elsewhere classified) for further adjustment because there were overlapping symptoms between unipolar depression and bipolar depression [29].

### Statistical analyses

In this study, the demographic characteristics and medical records in LHID included continuous data (such as age) and binary variables (such as sex, treatment status and comorbidity). They were all in reasonable range. The characteristics of the study population, which were represented by their age, sex, and comorbidities, were described using mean and percentage values. To examine the difference between the hepatitis and comparison cohorts, we used a *t* test to analyze age and a Chi square test to analyze sex and comorbidities. Furthermore, we calculated the incidence density of subsequent BD in both cohorts. The cumulative incidence curves for the 2 cohorts were evaluated using the Kaplan–Meier method. The log-rank test was used to test the difference between the incidence curves. To measure the risk of BD in the hepatitis cohort compared with the comparison cohort, we transformed all categorical variables into dummy variables and evaluated crude and adjusted hazard ratios (HRs) and 95% confidence intervals (CIs) by using Cox proportional hazard models. We also analyzed the risk of BD stratified by age, sex, and comorbidities. We used SAS Version 9.4 software (SAS Institute, Cary, NC, USA) to manage the data and conduct the statistical analyses. The incidence curves were plotted using R software (R Foundation for Statistical computing, Vienna, Austria). A 2-sided p < .05 reached the level of significance.

## Results

We selected a total of 48,215 patients with hepatitis in this study. As shown in Table 1, the types of hepatitis were HBV infection (71.5%), HCV infection (20.5%), and HBV/HCV coinfection (8.0%). The mean age of the hepatitis and comparison cohorts was approximately 46 years (SD: 15), and the male patients constituted the highest proportion (57.1%) of the patients in these cohorts. The proportions of comorbidities in the hepatitis cohort were significantly greater than those in the comparison cohort (all p < .001) (Table 1).

**Table 1:** Distribution of age, gender, and comorbidity between hepatitis infection and comparison cohort

	Hepatitis infection								Comparison N = 192,860		p value^†^
	Total (n = 48,215)		HBV (n = 34,459, 71.5%)		HCV (n = 9893, 20.5%)		Both (n = 3863, 8.0%)				
	N	%	N	%	N	%	N	%	N	%	
Age, year											0.99
20–34	12,799	26.6	11,028	32.0	1121	11.3	650	16.8	51,196	26.6	
35–49	16,568	34.4	13,023	37.8	2347	23.7	1198	31.0	66,272	34.4	
50–64	12,013	24.9	7447	21.6	3319	33.6	1247	32.3	48,052	24.9	
65+	6835	14.2	2961	8.59	3106	31.4	768	19.9	27,340	14.2	
Mean (SD)	46.5	(15.4)	43.4	(14.2)	55.8	(15.6)	51.0	(15.0)	46.3	(15.8)	0.001
Gender											0.99
Women	20,699	42.9	14,134	41.0	4863	49.2	1702	44.1	82,796	42.9	
Men	27,516	57.1	20,325	59.0	5030	50.8	2161	55.9	110,064	57.1	
Comorbidity											
Cirrhosis	28,450	59.0	18,803	54.6	6905	69.8	2742	71.0	23,111	12.0	< 0.001
Hypertension	12,542	26.0	6876	20.0	4331	43.8	1335	34.6	41,379	21.5	< 0.001
Hyperlipidemia	9671	20.1	6276	18.2	2501	25.3	894	23.1	27,681	14.4	< 0.001
Asthma	3026	6.28	1800	5.22	926	9.36	300	7.77	9114	4.73	< 0.001
CAD	6016	12.5	3107	9.02	2253	22.8	656	17.0	18,875	9.79	< 0.001
ALD	3216	6.67	1956	5.68	926	9.36	334	8.65	5304	2.75	< 0.001
Anxiety	3400	7.05	2081	6.04	976	9.87	343	8.88	8407	4.36	< 0.001
MDD	1615	3.35	967	2.81	464	4.69	184	4.76	3917	2.03	< 0.001

Chi square test

*CAD* coronary artery disease, *ALD* alcohol-related disorder, *MDD* major depressive disorders, *SD* standard deviation

^†^Total hepatitis infection versus comparison

**Fig. 1:**
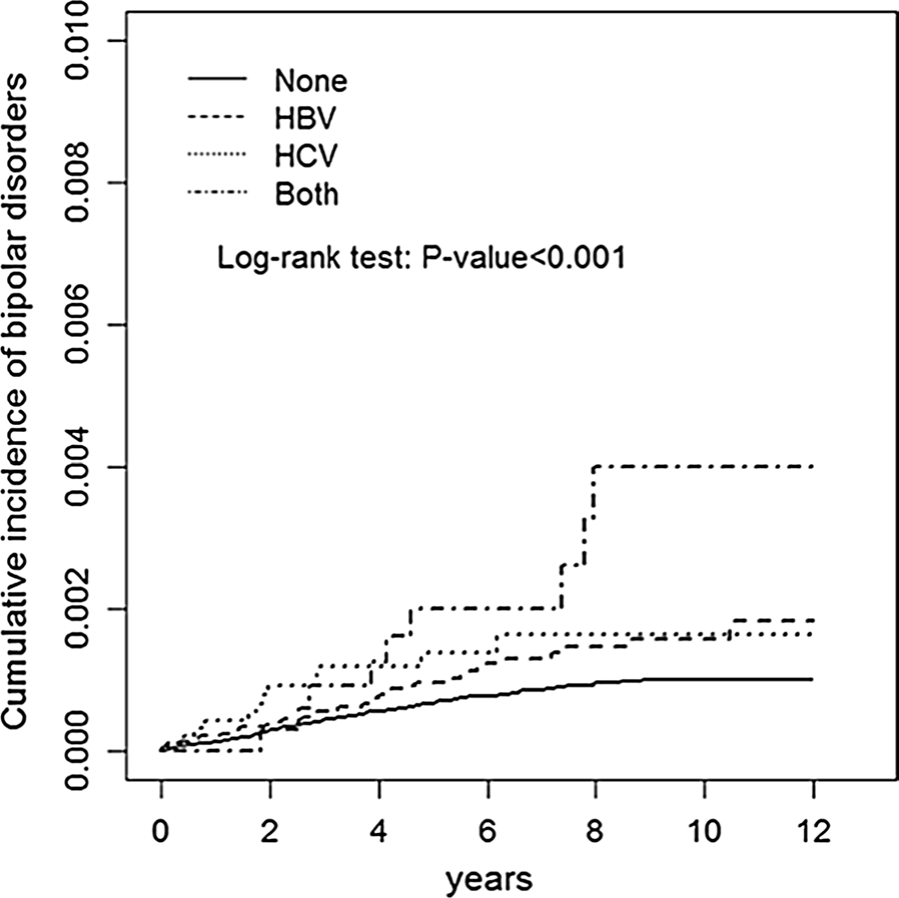
Cumulative incidence of bipolar disorders in patients with different hepatitis infection

We observed 136 and 58 BD occurrences in the comparison and hepatitis cohorts, respectively (Table 2). The incidence of BD in the comparison cohort was 1.14 per 10,000 person-years, whereas that in the hepatitis cohort was 2.04 per 10,000 person-years. Moreover, the incidence rates of BD were 1.79, 2.23, and 3.62 per 10,000 person-years in the HBV, HCV, and coinfection groups, respectively (Table 2). Figure 1 shows that the incidence curves for BD in the hepatitis cohort were significantly higher than those in the comparison cohort (log-rank test p < .001). After adjustment for BD-associated risk factors, patients with coinfection of HBV and HCV exhibited a significantly increased risk of BD compared with those in the comparison group (adjusted HR 2.16, 95% CI 1.06–4.41; p < .001) (Table 2). The results revealed a significantly higher risk for BD inpatients younger than 65 years old, female patients, and patients with the comorbidities of anxiety and major depressive disorder. We noted significantly lower risk for subsequent BD was the comorbidity of hyperlipidemia. In addition, we noted a study, conducted by Ayano et al. [27], showing sex difference among hepatitis infection, and having influence on the outcome. Hence, sex was a confounding factor in our study and we adjusted it in our study.

**Table 2:** Estimation of bipolar Incidence and hazard ratio by Cox proportional hazard models

	Event no	PY	%	HR (95% CI)	
				Crude	Adjusted
Hepatitis infection					
None	136	1,188,037	1.14	1.00	1.00
All	58	284,895	2.04	1.78 (1.31, 2.41)***	1.38 (0.96, 1.98)
HBV	37	206,332	1.79	1.56 (1.09, 2.25)*	1.27 (0.85, 1.90)
HCV	12	53,706	2.23	1.93 (1.07, 3.48)*	1.38 (0.73, 2.60)
Both	9	24,857	3.62	3.22 (1.64, 6.32)***	2.16 (1.06, 4.41)*
Age, year					
20–49	125	951,575	1.31	1.85 (1.04, 3.27)*	2.54 (1.34, 4.82)**
50–64	56	346,885	1.61	2.22 (1.21, 4.06)**	2.51 (1.35, 4.67)**
65+	13	174,472	0.75	1.00	1.00
Gender					
Women	104	635,836	1.64	1.53 (1.15, 2.02)**	1.37 (1.03, 1.83)*
Men	90	837,096	1.08	1.00	1.00
Comorbidity					
Cirrhosis					
No	135	1,168,092	1.16	1.00	1.00
Yes	59	304,840	1.94	1.66 (1.22, 2.26)**	1.23 (0.84, 1.80)
Hypertension					
No	151	1,177,342	1.28	1.00	1.00
Yes	43	295,590	1.45	1.10 (0.79, 1.55)	1.05 (0.69,1 .60)
Hyperlipidemia					
No	171	1,265,096	1.35	1.00	1.00
Yes	23	207,837	1.11	0.80 (0.52, 1.23)	0.54 (0.33, 0.87)*
Asthma					
No	181	1,410,513	1.28	1.00	1.00
Yes	13	62,419	2.08	1.55 (0.88, 2.72)	1.24 (0.69, 2.22)
CAD					
No	170	1,337,581	1.27	1.00	1.00
Yes	24	135,352	1.77	1.36 (0.89, 2.08)	1.13 (0.68, 1.88)
ALD					
No	185	1,434,621	1.29	1.00	1.00
Yes	9	38,311	2.35	1.71 (0.87, 3.34)	1.11 (0.56, 2.21)
Anxiety					
No	165	1,415,652	1.17	1.00	1.00
Yes	29	57,280	5.06	4.08 (2.75, 6.06)***	1.80 (1.15, 2.83)*
Major depressive disorders					
No	147	1,445,019	1.02	1.00	1.00
Yes	47	27,913	16.8	15.8 (11.4, 22.0)***	12.9 (8.85, 18.7)***

Multivariable analysis including age, sex, and comorbidities of cirrhosis, hypertension, hyperlipidemia, asthma, CAD, ALD, anxiety and major depressive disorders

*PY* person-years,  *%* Rate, per 10,000 person-years, *CAD* coronary artery disease, *ALD* alcohol-related disorder

* p < 0.05, ** p  < 0.01, *** p < 0.001

Table 3 presents the risk for BD stratified by age, sex, comorbidity, and types of hepatitis. Comparing the hepatitis cohort with the comparison cohort revealed that the HRs of BD significantly increased in patients aged more than 65 years (HR 4.49, 95% CI 1.25–16.2). Compared with the control group, a significantly increased risk for BD was observed in the female (HR 1.70, 95% CI 1.06–2.75) patients in the hepatitis group. In the HBV group, an increased risk of subsequent BD was noted among female patients (HR 1.87, 95% CI 1.13–3.10). In the HCV group, no association with the increased risk of subsequent BD was shown regardless of age, gender, and comorbidity. In both the HBV and HCV groups, an increased risk of subsequent BD was noted among patients aged more than 65 years (HR 16.3, 95% CI 2.83–93.5), and patients having any comorbidity (HR 2.28, 95% CI 1.09–4.76). In Table 2, crude HRs were the results of simple Cox regression, and adjusted HRs were the results of multiple Cox regression with fully adjustment. Table 3 shows the results of stratified analysis. We stratified by sub groups of each covariate, and calculated the HRs. Table 3 shows the results of stratified analysis. We stratified by sub groups of each covariate, and calculated the HRs. We have further modified Table 3. The adjusted HRs in total subjects (n = 48,215), HBV (n = 34,459), HCV (n = 9893) and Both HBV/HCV (n = 3863) compared with Comparison group (n = 192,860).

**Table 3:** Incidence and hazard ratio for bipolar disorders stratified by age, gender and comorbidity

	Comparison N = 192,860		Hepatitis infection											
			Total (n = 48,215)			HBV (n = 34,459)			HCV (n = 9893)			Both (n = 3863)		
	Event no	%	Event no	%	HR^†^ (95% CI)	Event no	%	HR^†^ (95% CI)	Event no	%	HR^†^ (95% CI)	Event no	%	HR^†^ (95% CI)
Age, year														
20–49	87	1.14	38	2.01	1.35 (0.86, 2.11)	28	1.81	1.30 (0.80, 2.10)	5	2.28	1.17 (0.45, 3.03)	5	3.83	2.19 (0.85, 5.62)
50–64	41	1.45	15	2.30	1.04 (0.53, 2.05)	8	2.05	0.98 (0.43, 2.21)	5	2.72	1.19 (0.44, 3.21)	2	2.58	1.02 (0.24, 4.46)
65+	8	0.56	5	1.64	4.49 (1.25, 16.2)*	1	0.77	2.12 (0.25, 17.7)	2	1.49	4.58 (0.83, 25.4)	2	4.95	16.3 (2.83, 93.5)**
Gender														
Women	73	1.43	31	2.49	1.70 (1.06, 2.75)*	24	2.80	1.87 (1.13, 3.10)*	4	1.47	1.02 (0.35, 2.92)	3	2.63	1.80 (0.54, 5.94)
Men	63	0.93	27	1.68	1.09 (0.65, 1.85)	13	1.08	0.76 (0.39, 1.46)	8	3.02	1.71 (0.77, 3.79)	6	4.46	2.42 (0.99, 5.96)
Comorbidity														
None	69	0.88	13	1.62	1.74 (0.96, 3.15)	11	1.63	1.74 (0.92, 3.31)	1	1.22	1.29 (0.18, 9.30)	1	2.38	2.54 (0.35, 18.3)
With any one	67	1.66	45	2.20	1.19 (0.81, 1.76)	26	1.87	0.95 (0.59, 1.52)	11	2.42	1.46 (0.77, 2.77)	8	3.87	2.28 (1.09, 4.76)*

%: Rate, per 10,000 person-years

* p < 0.05, ** p < 0.01, *** p < 0.001

^†^ Multivariable analysis including age, sex, and comorbidities of cirrhosis, hypertension, hyperlipidemia, asthma, coronary artery disease, alcohol-related illness, anxiety, and major depressive disorders

In Table 4, we demonstrated the association between antiviral drugs for hepatitis and the incidence of BD, revealing no association between antiviral drugs and increased risk of subsequent BD. We demonstrated the association between antiviral drugs for hepatitis and the incidence of BD, revealing no association between antiviral drugs and increased risk of subsequent BD. In Table 3, it shows incidence, and hazard ratio of bipolar disorders between patients with hepatitis infection with and without treatment (with propensity score matching). Table 4 shows estimation of bipolar hazard ratio by simple and multiple Cox proportional hazard models (with propensity score matching).

**Table 4:** Incidence, and hazard ratio of bipolar disorders between patients with hepatitis infection with and without treatment

Variables	N	Event	PY	%	Crude HR (95% CI)	Adjusted HR† (95% CI)
Hepatitis infection						
Without treatment	44,461	54	262,819	2.05	1.00	1.00
Only HBV treatment						
Lamivudine, telbivudine, adefovir, tenofovir, and entecavir	2151	2	11,820	1.69	0.82 (0.20, 3.36)	0.85 (0.21, 3.53)
Only HCV treatment						
Ribavirin, interferon	1081	2	6623	3.02	1.48 (0.36, 6.05)	1.29 (0.31, 5.37)
Both HBV&HCV treatment	522	0	3634	0.00	–	–

*PY* Person-years,  *%* rate, per 10,000 person-years

^†^ Multivariable analysis including age, sex, and comorbidities of cirrhosis, hypertension, hyperlipidemia, asthma, coronary artery disease, alcohol-related illness, anxiety, and major depressive disorders

### Discussion

#### Key findings

In this study, we demonstrated that HBV or HCV infection may be associated with subsequent BD, and, to the best of our knowledge, we demonstrated their correlation for the first time by using a matched population-based cohort and 10-year follow-up period. The major findings of our study suggest a higher incidence of subsequent BD among patients with HBV/HCV coinfection. Also, we demonstrated the comorbidities related to the subsequent occurrence of BD, including hyperlipidemia, anxiety, and major depressive disorder. Anxiety was frequently comorbid with BD [28]. Patients with major depression may have unrecognized BD [30], and depressive symptoms were noted among the course of BD [31]. On the other hand, evidence showed patients with chronic hepatitis B and C had psychosocially impaired their mental health and daily life [32]. As a result, those conditions were associated with subsequent occurrence of BD. In addition, we noted hyperlipidemia had decreased risk for subsequent BD. We considered this may be associated with selection bias in our study. Mood stabilizer and atypical antipsychotics were used for the treatment of BD [33]. The metabolic syndrome, including hyperlipidemia, was reported as the adverse effect of the treatment [34]. As a result, patients with BD under those drugs treatment may have improved clinical condition but have the adverse effect of hyperlipidemia.

We noted that female patient with HBV infection was associated with the subsequent occurrence of BD. To the best of our knowledge, we hypothesized that the mechanism was possibly related to the interaction between sex hormone and the disease activity of HBV infection. Evidence shows that HBV infection is characterized by its lifelong persistence and inflammation [35]. In addition, acute liver failure (ALF) was noted to be among the courses of chronic liver inflammation [36]. Moreover, proinflammatory cytokines such as TNF-α may affect the brain by impairing the permeability of the blood brain barrier and neuroinflammation, particularly under the condition of ALF [37, 38]. In addition, studies have revealed that inflammation in the brain was associated with developing mood disorders, including BD, and that TNF-α was a critical cytokine in pathophysiology [39–41]. On the other hand, one study showed that sex hormones were associated with the disease activity of HBV infections [42]. Although estrogen was reported to have a protective effect on the progression of HBV infection [43], estrogen receptor gene polymorphisms in a Chinese population may be associated with occurrences of ALF [44]. As a result, female patients with HBV demonstrate an increased risk of subsequent BD.

It has been shown elsewhere that the anti-HCV treatment (ribavirin and interferon) are related to the development of depression [21]. In our study, we demonstrated the association between HBV/HCV and subsequent BD. When we further control the effects of anti-viral treatments to HBV or HCV infections (anti-HBV treatment: lamivudine, telbivudine, adefovir, tenofovir, and entecavir or anti-HCV treatment: ribavirin, interferon), we found no association between HBV or HCV treatment and increased risk of subsequent BD (Table 4). Hence, we considered the association between HBV/HCV and subsequent BD may be attributed to the HBV/HCV infection/inflammation itself rather than the effect of treatment. However, future mechanistic studies are warranted.

In addition, our statistically matched study, including age, gender, and comorbidity, demonstrated increased risk of subsequent BD in patients having concurrent HBV and HCV infections. Our results reveal that this condition was associated with patients aged more than 65 years and having any comorbidity. HBV and HCV have been reported to be associated with CNS involvement through neuroinflammation [37, 45]. HBV/HCV coinfection is prone to involve more advanced and progressive liver damage than does monoinfection [46]. Although treatment for HCV dominance in HBV/HCV coinfection can achieve HCV clearance, HBV reactivation has been noted [3, 47]. The reactivation of HBV could induce immune-mediated inflammatory reactions [48, 49], which are superimposed with the underlying chronic inflammation associated with HBV and HCV conditions. Hence, the inflammatory process was augmented, consequently increasing the risk of BD. In addition, the aforementioned studies have indicated that the ALF of HBV infection could impair the integrity of the blood–brain barrier [37, 38], and HCV could infect brain endothelial cells [50], induce microglial activation [51], and adversely interact with brain astrocytes [52]. We suppose that the adverse influence of HBV and HCV cause the brain to be more vulnerable to neuroinflammation and HCV replication. Hence, the risk of BD increased more in HBV/HCV coinfection than in HBV or HCV infection. On the other hand, studies had shown the association between aging and neuroinflammation [53], and we considered this condition may make elder patients with HBV/HCV coinfection more vulnerable to subsequent BD [54]. Hence, we suppose that in patients with HBV/HCV coinfection, those aged more than 65 years may raise further concern about the increased risk of subsequent BD. Moreover, HCV was reported having comorbidities involving in systemic inflammation [55, 56] and may therefore increase the risk of subsequent BD.

#### Limitations

To the best of our knowledge, this is the first population-based study designed to investigate the association between viral hepatitis and BD. We employed a matched case–control design and a population-based cohort of patients with viral hepatitis and adequate controls as well as a sufficient number of comorbidities, thus further strengthening the study. However, limitations inherent to the use of claims databases were encountered. First, the diagnosis of hepatitis in the NHIRD was based on ICD-9-CM codes; therefore, the severity of hepatitis as a risk factor for developing BD was not explored. Also, we mark the temporal association by the chronological order, so we could not confirm the effect or the causal relationship between hepatitis infection and BD. Additionally, considering the possibility of reactivation, we included hepatitis carrier, ICD-9-CM codes of V02.61 and V02.62, in our study, and this management could affect the result revealed in our study. Nevertheless, in our study design, we conducted a 10-year follow-up to observe the long-term influence of HBV or HCV infection on the subsequent development of BD. Second, the association was evaluated according to the chronological order in which these two diseases were diagnosed. Whether the patients previously had BD-related symptoms without being diagnosed with BD was unknown. Third, information on numerous demographic variables, including socioeconomic status and family history, was unavailable; such information may provide useful data regarding factors associated with viral hepatitis and BD. Forth, BD was reported having early presentation in lifetime prevalence [57]. Excluding patients younger than 20 years old in this study may limit the application. However, studies had shown that attention deficit hyperactivity disorder was often comorbid and easily confused with BD in younger age [58]. Therefore, our study used more than 20 years old instead and demonstrated on the long-term relationship between HBV/HCV and BD. Furthermore, some immune-related diseases, such as rheumatoid arthritis [17], were reported having association with subsequent BD. Hence, those diseases could be confounding factors. On the other hand, exclusion of depression and anxiety may have influence on the diagnosis of BD, and therefore influence the generalizability of our study. Forth, although our study sample comprised an adequate number of patients in the HBV/HCV and control groups as well as an adequate number of comorbidities, our findings were demonstrated only in this studied population; we could not validate the generalizability of the results to other populations. Finally, although we also provided the propensity score matching for hepatitis versus no-hepatitis patients (Additional file 1: Tables S1 and S2), there is no significance for the casual relationship of viral hepatitis and BDs, it may be due to the reason that if we deliberately adjust the control cohort for hypertension, hyperlipidemia, asthma, CAD, ALD, anxiety and MDD, then we may overlook the true risk for BDs in HBV, HCV or HBV/HCV patients, because the new control cohort consists of subjects with relatively higher risk. Due to the association between viral hepatitis and BDs, and the significant role of neuronal dysfunction are strongly suggested in BDs, we also plan to confirm the causal relationship of viral hepatitis and BDs, and the underlying mechanisms by using the human induced pluripotent stem cells from HBV/HCV patients and differentiated the neurons that are responsible for the phenotype of BD [59].

## Conclusions

The findings suggest that HBV/HCV infection may be associated with increased risk of subsequent BD. Additional prospective clinical and basic studies on the relationship between hepatitis and BD are warranted.

## Additional file

**Additional file 1: Table S1.** Distribution of age, gender, and comorbidity between hepatitis infection and comparison cohort with propensity score matching. **Table S2.** Estimation of bipolar Incidence and hazard ratio by Cox proportional hazard models with propensity score matching.

## Abbreviations

ALF: Acute liver failure
BD: Bipolar disorder
CIs: Confidence intervals
HRs: Hazard ratios
HBV: Hepatitis B virus
HCV: Hepatitis C virus
ICD-9-CM: International Classification of Diseases, Ninth Revision, Clinical Modification
IRB: Institutional Review Board
LHID: Longitudinal Health Insurance Database
NHI: National Health Insurance
NHRI: National Health Research Institutes
