# Supplementary material for: Association of viral hepatitis and bipolar disorder: a nationwide population-based study
Source: J Transl Med. 2018 Jun 22;16:173. doi: 10.1186/s12967-018-1542-3 (PMC6013873; doi:10.1186/s12967-018-1542-3)
Supplement: Supplementary file 1 — Additional file 1: Table S1. Distribution of age, gender, and comorbidity between hepatitis infection and comparison cohort with propensity score matching. Table S2. Estimation of bipolar Incidence and hazard ratio by Cox proportional hazard models with propensity score matching. [file 12967_2018_1542_MOESM1_ESM.docx]

Table S1. Distribution of age, gender, and comorbidity between hepatitis infection and comparison cohort with propensity score matching

|  | Hepatitis infection  N=41128 | | Comparison N=41128 | |
| --- | --- | --- | --- | --- |
|  |  | |  | |
|  | n | % | n | % |
| Age, year |  |  |  |  |
| 20-34 | 9261 | 22.5 | 9226 | 22.4 |
| 35-49 | 13931 | 33.9 | 14004 | 34.1 |
| 50-64 | 11309 | 27.5 | 11367 | 27.6 |
| 65+ | 6627 | 16.1 | 6531 | 15.9 |
| Mean (SD) | 48.0 | 15.5 | 48.1 | 15.5 |
| Gender |  |  |  |  |
| Women | 17344 | 42.2 | 17623 | 42.9 |
| Men | 23784 | 57.8 | 23505 | 57.2 |
| Comorbidity |  |  |  |  |
| Cirrhosis | 21363 | 51.9 | 21363 | 51.9 |
| Hypertension | 11778 | 28.6 | 12280 | 29.9 |
| Hyperlipidemia | 9361 | 22.8 | 10514 | 25.6 |
| Asthma | 2661 | 6.47 | 2869 | 6.98 |
| CAD | 5759 | 14.0 | 6053 | 14.7 |
| ALD | 2604 | 6.33 | 2748 | 6.68 |
| Anxiety | 2904 | 7.06 | 3199 | 7.78 |
| MDD | 1359 | 3.30 | 1471 | 3.58 |

Chi-square test. †Total hepatitis infection vs. comparison. Abbreviation: CAD: coronary artery disease; ALD: alcohol-related disorder; MDD: Major depressive disorders; SD: standard deviation

Table S2. Estimation of bipolar Incidence and hazard ratio by Cox proportional hazard models with propensity score matching

|  |  | |  | HR (95% CI) | |
| --- | --- | --- | --- | --- | --- |
|  | Event no | PY | % | Crude | Adjusted |
| Hepatitis infection (n=50) |  |  |  |  |  |
| None | 40 | 246625 | 1.62 | 1.00 | 1.00 |
| HBV | 31 | 170410 | 1.82 | 1.12(0.70, 1.80) | 1.12(0.70, 1.80) |
| HCV | 12 | 46881 | 2.56 | 1.56(0.82, 2.98) | 1.53(0.79, 2.95) |
| Both | 7 | 21057 | 3.32 | 2.11(0.95, 4.71) | 1.87(0.83, 4.20) |

PY: person-years; Rate, per 10000 person-years

multivariable analysis including age, sex, and comorbidities of cirrhosis, hypertension, hyperlipidemia, asthma, CAD, ALD, anxiety and major depressive disorders

Abbreviation: CAD: coronary artery disease; ALD: alcohol-related disorder

*p<0.05, **p<0.01, ***p<0.001
